# Psychosocial Predictors of Postpartum Posttraumatic Stress Disorder in Women With a Traumatic Childbirth Experience

**DOI:** 10.3389/fpsyt.2018.00348

**Published:** 2018-07-31

**Authors:** Mark A. van Heumen, Martine H. Hollander, Maria G. van Pampus, Jeroen van Dillen, Claire A. I. Stramrood

**Affiliations:** ^1^Department of Obstetrics and Gynecology, Radboud University Medical Center, Nijmegen, Netherlands; ^2^Department of Obstetrics and Gynecology, Onze Lieve Vrouwe Gasthuis, Amsterdam, Netherlands; ^3^Department of Obstetrics and Gynecology, University Medical Center Utrecht, Utrecht, Netherlands

**Keywords:** posttraumatic stress disorder, childbirth, traumatic experience, predictors, social support, coping, prevention

## Abstract

**Objective:** To analyze the predictive value of antepartum vulnerability factors, such as social support, coping, history of psychiatric disease, and fear of childbirth, and intrapartum events on the development of symptoms of postpartum posttraumatic stress disorder (PP-PTSD) in women with a traumatic childbirth experience.

**Materials and methods:** Women with at least one self-reported traumatic childbirth experience in or after 2005 were invited to participate through various social media platforms in March 2016. They completed a 35-item questionnaire including validated screening instruments for PTSD (PTSD Symptom Checklist, PCL-5), social support (Oslo social support scale, OSS-3), and coping (Antonovsky's sense of coherence scale, SoC).

**Results:** Of the 1,599 women who completed the questionnaire, 17.4% met the diagnostic criteria for current PTSD according to the DSM-5, and another 26.0% recognized the symptoms from a previous period, related to giving birth. Twenty-six percent of the participating women had received one or more psychiatric diagnoses at some point in their life, and five percent of all women had been diagnosed with PTSD prior to their traumatic childbirth experience. Women with poor (OR = 15.320, CI = 8.001–29.336), or moderate (OR = 3.208, CI = 1.625–6.333) coping skills were more likely to report PP-PTSD symptoms than women with good coping skills. Low social support was significantly predictive for current PP-PTSD symptoms compared to high social support (OR = 5.557, CI = 2.967–7.785). A predictive model which could differentiate between women fulfilling vs. not fulfilling the symptom criteria for PTSD had a sensitivity of 80.8% and specificity of 62.6% with an accuracy of 66.5%.

**Conclusions:** Low social support, poor coping, experiencing “threatened death” and experiencing “actual or threatened injury to the baby” were the four significant factors in the predictive model for women with a traumatic childbirth experience to be at risk of developing PP-PTSD. Further research should investigate the effects of interventions aimed at the prevention of PP-PTSD by strengthening coping skills and increasing social support, especially in women at increased risk of unfavorable obstetrical outcomes.

## Introduction

For a long time, childbirth has been regarded by professionals as a positive experience for the mother, but in the past two decades there has been increasing attention in research and clinical practice for women with a negative or even traumatic childbirth experience. In some cases this experience can lead to a postpartum posttraumatic stress disorder (PP-PTSD) (1). Two recent systematic reviews estimated the prevalence of PP-PTSD at 3.1 and 4.0%, in unselected or community samples, respectively (2, 3). A third systematic review suggested a prevalence of 4.9% for PTSD in the first 6 months after childbirth in women without a prior history of PTSD (4). Additionally, 9.6% of the women in this study had at least some symptoms of PP-PTSD. Literature about women with a self-reported traumatic childbirth experience is scarce, despite a reported prevalence of 9.1–45.5%. Frequently mentioned attributions of the trauma are lack or loss of control, breeched expectations about giving birth, perception of inadequate intrapartum care, and the level of obstetric intervention experienced during birth (5–7).

The diathesis-stress model is frequently used to understand the risk factors for developing PP-PTSD. This approach implies that the development of PP-PTSD depends on a combination of the degree of antepartum vulnerability, the events during delivery and postpartum factors (8). A previous history of psychiatric disease, depression during the current pregnancy, fear of childbirth and medical complications during childbirth have previously been identified to contribute to antepartum vulnerability for PP-PTSD. Operative birth (unplanned cesarean section and instrumental delivery), dissociation, lack of support by medical staff, and loss of control during delivery are also known contributors to the development of PP-PTSD, as well as poor coping after childbirth (9). Many of these factors are also consistent with the known risk factors for a traumatic childbirth experience (7, 10).

Being able to identify antepartum vulnerability factors and events during delivery could be helpful in designing future interventions aimed at reducing the risk of women experiencing giving birth as traumatic and/or preventing the development of PP-PTSD (symptoms). To date, there are only three studies that analyzed risk factors for developing PP-PTSD in women with a traumatic childbirth experience based on criterion A of the Diagnostic and Statistical Manual of Mental Disorders (DSM-IV) (7, 10–12). Two studies aimed to provide predictors for the development of PP-PTSD that could be used for the construction of a screening tool or intervention strategy (7, 10). The most recent study of Dikman-Yildiz et al. (12) investigated the different trajectories of birth-related PTSD and the predictors of each trajectory. This study showed that the development of PP-PTSD in women with a traumatic childbirth experience could be divided in four trajectories: resilience (61.9%), recovery from PTSD (18.5%), chronic PTSD (13.7%), and delayed PTSD (5.8%).

Recently, a systematic review about prevention of traumatic childbirth experiences and PP-PTSD found that to date there is no study investigating primary prevention of a traumatic childbirth experience. A few studies with insufficient level of evidence have investigated the secondary prevention of PP-PTSD or a traumatic childbirth experience (13). Being able to identify antepartum vulnerability factors and predisposing events during delivery could be helpful in designing future interventions aimed at reducing the risk of women experiencing childbirth as traumatic and/or preventing the development of PP-PTSD (symptoms).

The first objective of the current study was to analyze the role of antepartum vulnerability factors in the development of PP-PTSD symptoms in women with a traumatic childbirth experience, such as social support, sense of coherence, a history of psychiatric disease, and fear of childbirth, in addition to events during delivery. The second objective of this study was to make a predictive model using these antepartum vulnerability factors and factors during delivery, which could differentiate between women who have experienced childbirth as traumatic and who are more likely to develop PP-PTSD symptoms. That way, antepartum vulnerability factors and factors during delivery could be identified and future interventions designed to prevent PP-PTSD symptoms could be designed.

## Methods

### Setting/research design

A retrospective study was carried out among women with at least one self-reported traumatic childbirth experience in the Netherlands between 2005 and 2016. In the Netherlands, maternity care is organized differently from many other high income countries. It is divided into two levels of care: healthy women with a low-risk pregnancy are cared for by independent community midwives during pregnancy and childbirth, while women with (a higher risk of) complications during current or previous pregnancies or women with specific healthcare problems receive care from an obstetrician in a hospital setting. The recommended level of care is based on national guidelines (14).

### Participants

Participants were eligible for inclusion in the study if they were 18 years or older, if they had a history of at least one traumatic childbirth experience in the Netherlands between 2005 and 2016 and if they could read and write in Dutch.

### Procedure

The participants were invited to fill out an online questionnaire if they had an affirmative response to the question “Did you have a traumatic birth experience?” The questionnaire was accessible through SurveyMonkey^1^ for a period of 3 weeks in March of 2016. Participants were recruited through a designated website (www.traumatischebevalling.nl), and a Facebook page and Twitter account created for the purpose of the study. Various Dutch support groups, like the HELLP Syndrome Foundation, Traumatic Childbirth & Postpartum Depression, Birth Movement and Association for Parents of Incubator Babies and two professional associations [Royal Dutch Association Of Midwives (KNOV) and Dutch Association of Obstetrics and Gynecology (NVOG)], shared the questionnaire on their online pages at our request. Ethical approval for this study was deemed unnecessary by the medical ethics committee of the Radboud University Nijmegen.

Data were collected online and transferred to SPSS version 22 (IBM Corporation Inc., Armonk, NY, USA). Questionnaires with the same IP address (multiple entries) or inconsistent answers (e.g., planned cesarean section during home birth) were excluded from the data set. The results of the first part of the dataset, which concerned women's attributions regarding their traumatic childbirth experience and what their caregiver or they themselves could have done to prevent the trauma, have already been published (15). To be included in the current article, participants had to fill out the complete questionnaire up until and including the last item about Sense of Coherence, which was one of the variables in our study.

### Measurements

The questionnaire consisted of items regarding demographic information of the participants, attributions of their traumatic childbirth experience, medical details, and various risk factors for PP-PTSD known from literature. The questionnaire also contained four psychological measurement tools (see details below). The first draft of the questionnaire was reviewed by two parties: members of the Childbirth and Psychotrauma Research (CAPTURE) group of the hospital OLVG in Amsterdam, the Netherlands and the committee for patient communication of the NVOG.

Presence of criterion A of the DSM-5 (16) was determined through questions about the threat to participants' own life or the life of others and actual or threatened serious injury to self or others. Threat to participants' physical integrity, which was included in criterion A1 of the DSM-IV but left out of the DSM-5, was determined in order to compare criterion A1 of the DSM-IV with criterion A of the DSM-5.

The Posttraumatic stress disorder CheckList (PCL-5) was developed to measure symptoms of posttraumatic stress disorder according to the DSM-5. The participants were asked to fill out the checklist in relation to their traumatic childbirth experience. The PCL-5 consists of 20 questions corresponding with 20 symptoms of category B (re-experiencing), C (avoidance), D (negative thoughts and feelings), and E (trauma-related arousal and reactivity) of the DSM-5. All statements were followed by five-point Likert scales (range zero to four). A score of two or higher was considered clinically relevant. The criterion was met for category B and C when there was at least one clinically relevant symptom in each category. Two clinically relevant symptoms were needed for category D and E (17). If all four criteria were met in combination with the A-criterion, current PP-PTSD was considered likely.

The Sense of Coherence (SoC) is a validated questionnaire with 13 items measuring the way in which a person sees the world as comprehensible, manageable, and meaningful (18). Strong SoC is indicative of effective coping strategies. The 13 items are rated on a seven-point Likert scale, with a total possible score between 13 and 91. The data from the questionnaire were used as continuous variables, but also divided into the three groups used in literature pertaining to SoC and childbirth (19, 20). The groups were defined as follows: a score under 60 points was defined as a low SoC score, a score between 61 and 75 was a moderate score and 76 or higher was a high score.

The social support of a participant was measured with the Oslo Social Support Scale (OSS-3). The OSS-3 is a validated three-item questionnaire with questions about the number of people a participant can count on, how much interest people are showing regarding the participant and how easily the participant could get help from neighbors. The total possible score of the OSS-3 ranges between 3 and 14 A score of 3 to 8 indicates poor support, a score of 9 to 11 means moderate support and a score of 12 to 14 signifies high support (21, 22).

Fear of childbirth was measured on a ten-point scale. Measurement of fear of childbirth with a ten-point scale has been validated compared to the W-DEQ questionnaire, which is a validated tool for measuring fear of childbirth. A threshold of 5.0 for a positive score has been demonstrated in literature to have a sensitivity of 97.8% and a specificity of 65.7% in comparison with a score of ≥100 on the W-DEQ questionnaire, signifying extreme fear of childbirth (23).

### Data analysis

The characteristics of women with a traumatic childbirth experience were summarized by descriptive statistics. Chi square tests were used to compare the characteristics of different groups. Continuous variables (fear of childbirth, Oslo Social Support and Sense of Coherence) were not distributed normally. Therefore, Mann-Whitney U-tests were used for comparative analyses. Logistic regression analyses were used for antepartum vulnerability factors (Sense of Coherence, social support, fear of childbirth, parity, age), factors during delivery (mode of delivery, criterion A of the DSM-5, including threat to physical integrity, and caregiver during delivery). There were no postpartum factors used in our analyses. The ordinal groups for the Sense of Coherence-, Oslo Social Support- and Fear of childbirth analyses were only used in univariable logistic regression analyses. Univariable logistic regression analyses were performed to estimate odds ratios and their 95% confidence intervals for factors associated with symptoms of PP-PTSD (meeting all criteria B, C, D, and E for PTSD on the PCL-5). The same analyses were done for women who were treated for PP-PTSD and/or received a diagnosis of PP-PTSD from a psychologist, psychiatrist or general practitioner. A significance level of ≤ 0.05 was used. A predictive model was created with a multivariable logistic regression analysis, including only those variables that were statistically significant in univariable logistic regression, while using a backward likelihood ratio method and a logit function (entry −0.05; removal-0.10). This was done to establish predictive factors and determine their respective weight in predicting PP-PTSD. The Nagelkerke R^2^, which only gives a relative measure of R^2^ in logistic regression analysis, was used to evaluate the predictive power of the different models (24). The maximum value of the Youden index (J = sensitivity + specificity −1) was used to find the point at which the cutoff value of the formula reached optimal sensitivity and specificity, when sensitivity and specificity are given equal weight (25).

## Results

**Figure 1:**
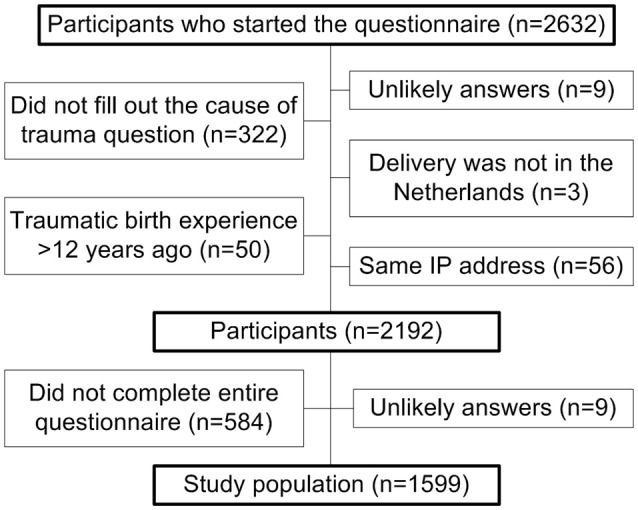
Flowchart of questionnaires excluded from the study.

A total of 2,634 questionnaires were filled out during a 3 week period in March of 2016. After removal of all questionnaires which did not meet the inclusion criteria for the first article published (15), 2192 questionnaires remained. An extra 584 participants were excluded from this current study, because they did not complete the questionnaire up until and including the last item about Sense of Coherence, which was not required for the previous article (Figure 1). A total of 1,599 questionnaires remained after exclusions.

The study population of women with a traumatic childbirth experience was compared to Dutch national data on all childbearing women. The study participants differed significantly from the national data for the same characteristics as published in the article by Hollander et al. (15): lower parity at time of traumatic childbirth experience, older age during childbirth, fewer deliveries between 37–42 weeks, fewer women of non-Dutch ethnicity, more unplanned cesarean sections, fewer planned cesarean sections, fewer spontaneous vaginal deliveries, and more referrals to a different level of care during pregnancy and delivery.

The basic characteristics of the participants who were excluded from the existing dataset of Hollander et al. (15) because they did not complete the entire questionnaire (*n* = 584) did not differ significantly from the participants that were included, except for their response to the DSM-A criterion: the 584 excluded women less often reported a threat to their own life (24.8 vs. 29.7%, *p* = 0.023), actual or threatened serious injury (26.3 vs. 30.8%, *p* = 0.040) and a threat to their physical integrity (28.3 vs. 38.9%, *p* < 0.001) compared to the women included in the analyses in the current article (*n* = 1599).

### Percentage of women meeting DSM criteria for PTSD

A majority of the participants (83.1%) experienced a traumatic childbirth according to criterion A1 of the DSM-IV, which included experienced threats to physical integrity, while 75.0% of the women met criterion A of the DSM-5. Women who met criterion A of the DSM-5 were significantly more likely to have been diagnosed with PP-PTSD by their general practitioner or a psychiatrist (18.8 vs. 8.6%, *p* = 0.003) compared to women meeting criterion A1 of the DSM-IV who did not meet criterion A of the DSM-5. They were, however, not more likely to get treatment for PTSD (21.7 vs. 14.8%, *p* = 0.072) than women meeting criterion A1 of the DSM-IV who did not meet criterion A of the DSM-5.

Table 1 gives an overview of the percentages of women meeting criteria A, B, C, D, and E. The percentages of women meeting criterion B through E ranged between 39.8 and 54.2%. A total of 17.4% (*n* = 278) of participants met all criteria (A, B, C, D, and E) based on the DSM-5, whereas 4.1% (*n* = 65) of participants fulfilled criteria B, C, D, and E but missed criterion A of the DSM-5. Of these 65 participants, 40.0% (26/65) would have met criterion A1 based on the DSM-IV, meaning that 26 women in this study were deemed not to have PP-PTSD by DSM V criteria, where they would have qualified according to DSM-IV criteria.

**Table 1:** The prevalence of criteria A, B, C, D, and E for PTSD among the participants.

**Characteristics (*n* = 1599)**	**Participants *n* (%)**
**CRITERION A**	
DSM-IV (A1)^a^	1328 (83.1)
DSM-5 (A)^b^	1200 (75.0)
**PTSD CHECKLIST (PCL-5)**	
Criterion B (Re-experiencing)	866 (54.2)
Criterion C (Avoidance)	651 (40.7)
Criterion D (Negative thoughts and feelings)	727 (45.5)
Criterion E (Trauma-related arousal and reactivity)	636 (39.8)
Criterion BCDE	343 (21.5)
Criterion ABCDE (DSM-5)	278 (17.4)
**RECOGNITION OF SYMPTOMS PCL-5**	
Recognition^c^	415 (26.0)

a *Diagnostic and Statistical Manual of Mental Disorders, American Psychiatric Association, 4th edition (1994)*.

b *Diagnostic and Statistical Manual of Mental Disorders, American Psychiatric Association, 5th edition (2013)*.

c *“I recognize these symptoms from earlier, these had to do with my traumatic childbirth experience” (PCL-5)*.

Participants were asked if they recognized the symptoms on the PCL-5 questionnaire from earlier, in order to identify women who had experienced symptoms of PP-PTSD in the past, but did not have symptoms at the time of completing the questionnaire. Twenty-six percent of the participants recognized these symptoms from an earlier period related to the traumatic childbirth experience.

### Antepartum vulnerability factors

The antepartum vulnerability factors of the participants are shown in Table 2. Sense of Coherence had a mean of 61.9 points for the study population. With a cut-off value of 60 or less for low coping abilities, it was found that almost half of the sample had poor coping abilities (44.6%), whereas 16.8% of participants had good coping abilities. A mean of 4.2 was found on a ten-point scale for fear of childbirth. The OSS-3 had a mean of 10.5 on a 14-point scale and showed that 19.4% of participants had poor social support. Twenty-six percent of the participating women had at some point in their lives received a psychiatric diagnosis unrelated to pregnancy or childbirth, and of all women, five percent had received a diagnosis of PTSD following a trauma other than the traumatic childbirth experience.

**Table 2:** Psychosocial factors in the study population.

**Antepartum factors (*n* = 1599)**	**Participants *n* (%) or mean {SD}**
**SENSE OF COHERENCE (13-91)**^a^	
Low sense of coherence	713 (44.6)
Moderate sense of coherence	617 (38.6)
High sense of coherence	269 (16.8)
Mean sense of coherence	61.9 {13.1}
**FEAR OF CHILDBIRTH (1-10)**	
Mean fear of childbirth	4.2 {2.3}
**OSLO SOCIAL SUPPORT (OSS-3) (3-14)**^b^	
Poor support	311 (19.4)
Moderate support	699 (43.7)
Strong support	589 (36.8)
Mean OSS-3	10.5 {2.3}
**HISTORY OF PSYCHIATRIC DISEASE**	
History of psychiatric disease	420 (26.3)
Depression	223 (13.9)
Posttraumatic stress disorder	80 (5.0)
Anxiety	118 (7.4)
Personality disorder	31 (1.9)
Other	84 (5.3)

a *Cut–off score Sense of Coherence: Low Sense of Coherence corresponds to 13–60 points, moderate Sense of Coherence 61–75 points, high Sense of Coherence 76–91 points*.

b *Cut-off score Oslo Social Support: Poor support corresponds to 3–8 points, moderate support to 9–11 points, strong support 12–14 points*.

### Predictors of a diagnosis of PP-PTSD or treatment for PTSD

Women who had a low sense of coherence were more often diagnosed with PP-PTSD by a psychologist, psychiatrist or their general practitioner compared to women with a high sense of coherence (OR = 1.811, CI = 1.217–2.697, *p* = 0.003). However, low sense of coherence was not significantly associated with women who received treatment for PP-PTSD (OR = 1.247, CI = 0.875–1.777, *p* = 0.222).

Women who had poor social support were more often diagnosed with PP-PTSD, compared to those with strong social support (OR = 1.676, CI = 1.181–2.379, *p* = 0.004), although women who had poor social support were not treated for PP-PTSD significantly more often than women with strong social support (OR = 0.859, CI = 0.606–1.219, *p* = 0.859).

Women with a diagnosis of PP-PTSD (OR = 1.315, CI = 0.999–1.731, *p* = 0.051) or who received treatment for PP-PTSD (OR = 0.879, CI = 0.671–1.152, *p* = 0.349) were not significantly more likely to have a score above the cutoff of five points on the fear of childbirth scale than those without diagnosis of or treatment for PP-PTSD.

### Predictors of current PTSD symptoms

The associations between antepartum vulnerability factors or factors during delivery and reporting PP-PTSD symptoms at time of participation are shown in Table 3. Each of the components of criterion A of the DSM-5 is a significant predictor for meeting all of the criteria (B, C, D, and E). Threat to physical integrity, which was criterion A1 of the DSM-IV, was a significant predictor for current PP-PTSD symptoms (OR = 1.409, CI = 1.107–1.794, *p* = 0.005).

**Table 3:** Psychosocial factors or characteristics of delivery and their association with the occurrence of PP-PTSD symptoms^a^.

**Predictor**	**Odds ratio**	**95% confidence interval**
Age of trauma (years)	0.98	0.95–1.01
First delivery (Multiparous versus primiparous)	0.90	0.67–1.20
**CRITERION A (EXPERIENCED.)**		
Threatened death	2.00^*^	1.56–2.56
Threatened death baby	1.54^*^	1.20–1.97
Actual or threatened injury to self	1.36^*^	1.05–1.74
Actual or threatened injury to the baby	1.70^*^	1.33–2.16
Threat to physical integrity	1.41^*^	1.11–1.79
**CAREGIVER PREGNANCY**		
Midwife	0.76^*^	0.60–0.97
Obstetrician/Gynecologist	1.16	0.84–1.61
Referral	1.19	0.93–1.53
**CAREGIVER DELIVERY**		
Midwife	0.83	0.48–1.45
Obstetrician/ Gynecologist	1.22	0.96–1.55
Referral	0.85	0.67–1.09
**MODE OF DELIVERY**		
Vaginal delivery	0.92	0.72–1.18
Instrumental delivery	0.91	0.69–1.19
Planned cesarean section	1.72	0.83–3.54
Unplanned cesarean section	1.13	0.87–1.47
**SENSE OF COHERENCE (SOC)**		
Each extra point on SoC	0.92^*^	0.91–0.93
Moderate SoC^b^	3.21^*^	1.63–6.33
Low SoC^b^	15.32^*^	8.00–29.34
**OSLO SOCIAL SUPPORT SCALE (OSS-3)**		
Each extra point on OSS-3	0.75^*^	0.71–0.79
Moderate support^c^	1.92^*^	1.41–2.62
Poor support^c^	5.56^*^	3.97–7.79
**FEAR OF CHILDBIRTH**		
Each extra point for fear of childbirth	1.11^*^	1.06–1.17
Fear of childbirth^d^	1.63^*^	1.27–2.09
**HISTORY OF PSYCHIATRIC DISEASE**		
Any history of psychiatric disease	1.66^*^	1.28–2.14
Posttraumatic stress disorder	2.59^*^	1.63–4.12
Depression	1.76^*^	1.28–2.41
Anxiety	1.40	0.92–2.14
Personality disorder	2.71^*^	1.31–5.59

* *Significant at p ≤ 0.05*.

a *Symptoms of PP-PTSD are defined as women meeting all criteria B, C, D and E for PTSD on the PCL-5*.

b *Reference group consists of all the participants with a high sense of coherence*.

c *Reference group consists of all the participants with a strong support*.

d *A cut-off of five points was used on the fear of childbirth scale*.

Women with a low (OR = 15.320, CI = 8.001–29.336, *p* ≤ 0.001), or moderate SoC were more likely to report PP-PTSD symptoms than women with a high SoC (OR = 3.208, CI = 1.625–6.333, *p* = 0.001). Women with poor (OR = 5.557, CI = 2.967–7.785, *p* ≤ 0.001) or moderate social support were more likely to report PP-PTSD symptoms compared to women with high social support (OR = 1.921, CI = 1.407–2.623, *p* ≤ 0.001). A cutoff at five points on the fear of childbirth scale was significantly predictive for women reporting PP-PTSD symptoms at time of participation (OR = 1.633, CI = 1.273–2.094, *p* < 0.001).

Women with antepartum check-ups in primary care had a significantly lower chance of having PP-PTSD symptoms at time of participation (OR = 0.759, CI = 0.597–0.966, *p* = 0.025). Women with a history of depression (OR = 2.589, CI = 1.628–4.120, *p* < 0.001), or PTSD (OR = 1.756, CI = 1.285–2.405, *p* < 0.001) reported PP-PTSD symptoms significantly more often. Overall, age and parity at the time of the traumatic childbirth experience and mode of delivery were not significantly associated with current PP-PTSD symptoms.

### Toward a predictive model for PP-PTSD symptoms

A multivariable logistic regression was performed with the aim of proposing a model for predicting PP-PTSD symptoms in women with a traumatic childbirth experience and to determine their respective weight in predicting PP-PTSD. The proposed model with a Nagelkerke R^2^ (24) of 0.275 includes four predictors (Table 4): score on the Sense of Coherence (OR = 0.927, CI = 0.916–0.939, *p* ≤ 0.001), score on the OSS-3 (OR = 0.901, CI = 0.846–0.961, *p* = 0.001), experiencing “threatened death” (OR = 1.919, CI = 1.451–2.537, *p* < 0.001) and experiencing “actual or threatened injury to the baby” (OR = 1.493, CI = 1.137–1.960, *p* = 0.004).

**Table 4:** Variables found to contribute significantly to predicting the occurrence of PP-PTSD symptoms in a multivariable logistic regression analysis.

**Predictor**	**Odds ratio**	**95% confidence interval**
**CRITERION A (EXPERIENCED.)**		
Threatened death to self	1.92^*^	1.45–2.54
Actual or threatened injury to the baby	1.49^**^	1.14–1.96
**SENSE OF COHERENCE (SOC)**		
Each extra point of SoC	0.93^*^	0.92–0.94
**OSLO SOCIAL SUPPORT SCALE (OSS-3)**		
Each extra point of OSS-3	0.90^*^	0.85–0.96

* *Significant at p ≤ 0.001*.

** *Significant at p ≤ 0.05*.

A formula for a predictive model for postpartum women meeting the DSM-5 criteria for PTSD (BCDE) was built using the beta coefficient of the variables mentioned above and the constant of the multiple logistic regression analysis. This model has an area under the receiver operating characteristic (ROC) curve of 0.795 (CI = 0.769–0.821, *p* < 0.001). The optimum sensitivity of 80.8% and specificity of 62.6% of this predictive model was found at a threshold of −1.57, using the Youden index (25). A score above −1.57 in the formula corresponds to a high possibility of current PP-PTSD symptoms. The accuracy of this model is 66.5% with a threshold of −1.57.

Multiple logistic regression analyses of women with either a diagnosis by a psychologist, psychiatrist, or general practitioner of or treatment for PP-PTSD in the past, were done to decrease the chance of missing women who had recovered from their earlier PP-PTSD symptoms. Significant variables in the analysis for predicting a diagnosis of or receiving treatment for PP-PTSD had a Nagelkerke R^2^ of respectively 0.086 and 0.038. Combining women with either a diagnosis of PP-PTSD by one of these professionals, or treatment for PP-PTSD, or meeting the DSM-5 symptom criteria for PTSD (B, C, D and E) at time of participation in the study led to analyses with a Nagelkerke R^2^ of 0.154. Overall, the low Nagelkerke R^2^ of the three analyses shows that the predictors that were used are less able to distinguish women with a history of or treatment for PP-PTSD from those without a history of or treatment for PP-PTSD than using current PTSD as the outcome variable. This makes the predictors in these three models less relevant as target points for research on prevention of PP-PTSD.

## Discussion

The objective of this retrospective study was to identify antepartum vulnerability factors and predisposing factors during childbirth, for use as predictors in a predictive model for the development of PP-PTSD in women with a traumatic childbirth experience. This predictive model was made to determine the respective weight of the different factors in order to be able to develop a preventative approach. Lack of social support, low sense of coherence, experiencing “threatened death” and experiencing “actual or threatened injury to the baby” were the four significant contributing factors in our predictive model. The results of this study extend current knowledge of risk factors for PP-PTSD by identifying the most significant predictors for the development of PP-PTSD in women with a traumatic childbirth experience. The significant antepartum vulnerability factors in our predictive model could possibly be used as intervention points aimed at improving coping and social support, thereby either preventing the traumatic experience or mitigating its consequences.

A predictive model was constructed with an overall accuracy of 66.5% at a threshold of −1.57 and a sensitivity of 80.8% and specificity of 62.6%, based on women with symptoms of PP-PTSD. In a study by O'Donovan et al. (7), a model was constructed for predicting PP-PTSD status 4–6 weeks after a traumatic childbirth experience. Their model had an overall accuracy of 92.1% and a sensitivity of 52.2% and specificity of 99.2%. The overall accuracy of that model is higher than the model in our study. This can partially be explained by the fact that they investigated other variables in their study and used 14 other predictors in their model in addition to the predictors we have investigated in our study. Also, the lower prevalence of PP-PTSD in their study population (8.5 vs. 21.5% of women meeting criteria B, C, D, and E in our study) in combination with a higher specificity makes it difficult to compare both models on their level of accuracy (26).

In addition, our study found that the variables poor coping abilities, low social support, fear of childbirth, antepartum check-ups in secondary care (including referrals), a history of PTSD (due to trauma other than giving birth), or depression and meeting the DSM-5 A criterion for PTSD were significantly associated with meeting the DSM-5 criteria B, C, D, and E for PP-PTSD in a univariable analysis. These variables correspond with previous studies about risk factors for PP-PTSD (5, 7, 9). Psychosocial characteristics were stronger predictors of PP-PTSD than mode of delivery or caregiver during delivery. These results confirm the earlier suggestion of O'Donovan et al. (7) that psychosocial predictors play a more prominent role in development of PP-PTSD than medical events. Also the increasing individualism in the current society makes it that pregnant women are more dependent on themselves. This will make it more interesting to focus on the effect of interventions aimed at increasing social support in pregnant women.

A remark must be made regarding the prevalence of psychiatric disease, which would ideally be compared to a reference group. Comparison with the largest nationwide study investigating the prevalence of psychiatric disorders in the Netherlands (NEMESIS-2) proved to be unreliable, because of a difference in methods used. In the current study the prevalence of psychiatric diagnoses was based on asking participating women about conditions diagnosed by psychologists, psychiatrists or general practitioners. There is no information about the way these diagnoses were made. Prevalence in the nationwide study was based on the CIDI 3.0, a questionnaire about symptoms (and not diagnosis) of different psychiatric diseases, as assessed by an interviewer (27). This could explain the higher prevalence (41.0%) of psychiatric disease in NEMESIS-2 in comparison with our study (26.3%).

Ideally, we would be able to distinguish women with a traumatic delivery experience who will develop PP-PTSD from those who will not. The closest proxy for this in this retrospective study would be to group women experiencing symptoms at the time of participation (i.e., meeting DSM-5 criteria on the PCL-5) and women with previous but not current childbirth-related PTSD (i.e., following treatment and/or a PP-PTSD diagnosis) together. This model, however, proved to be weaker than a model based solely on women with present symptoms of PP-PTSD. One reason for the predictors in our study being less able to distinguish between those two groups could be that these predictors, such as coping style, might have improved over time through treatment, or that the participants' social support deteriorated through their suffering from symptoms of PP-PTSD. However, there is no study to date reporting on changes in psychosocial functioning after treatment for PP-PTSD (13).

In addition, it is possible that more assertive women or women with a more “objective” trauma were more likely to seek and receive treatment than women with higher antepartum vulnerability. The latter is in line with the results of the current study, in which women who experienced threatened death were significantly more likely to get treatment than women without this experience. It could possibly be that women with a more “objective” trauma have been taken more seriously by their mental health care providers and have been more likely to be offered treatment.

In this study it was decided to consider coping style as an antepartum factor, because it could serve as an important predictor for an antepartum predictive model and a possible target for interventions aimed at prevention, despite the postpartum role of coping in the development of PP-PTSD and the possibility of this changing during delivery and following trauma (9, 19). There is also no literature available about recent changes in coping abilities.

The reason for including the A criterion “threat to physical integrity” from the DSM-IV in the questionnaire was based on the transition from the DSM-IV to the DSM-5 during the time period under investigation, and the hypothesis that for many women, loss of control, lack of informed consent and not being treated respectfully was crucial in their attribution of the trauma, as demonstrated in the article by Hollander et al. (15). Indeed, of the 19% (65/343) of women who meet the DSM-5 criteria for PTSD (B, C, D and E) but not the DSM-5 A criterion, 40% (26/65) do meet the DSM-IV A1 trauma criterion. This warrants further reflection on the applicability of the definition of trauma to women at risk for PP-PTSD. This is particularly important, given the wide range of women who report experiencing the delivery of traumatic, which is partially dependent on the definition of trauma and how this is measured.

## Strengths and limitations

There are a number of limitations to the current study design. A consequence of the retrospective design of this study is that the questionnaires can only hypothesize about the psychosocial situation before delivery, such as social support and sense of coherence. Recent studies found that sense of coherence can change during pregnancy (19, 20). In addition, recall bias could play a role in the manner that women look back on childbirth and for example the degree of fear of childbirth they experienced during pregnancy. This could lead to under- or overestimating the effect of different predictors, depending on the effect of PP-PTSD on those predictors. Another limitation is that self-reported symptoms of PP-PTSD were used in the analysis of the predictors, instead of a diagnosis based on a structured interview. Furthermore, consideration should be given to the fact that more than half of the women (54.3%) filled out the questionnaire more than 2 years after their traumatic childbirth experience, which may have led to missing women with PP-PTSD in remission. Finally, 26.0% of participants declared that they recognized the symptoms from the PCL-5 from an earlier period and that those symptoms had to do with the traumatic childbirth experience. It is possible that women with severe symptoms of PP-PTSD were more likely to fill out the questionnaire, leading to an overestimation of the effect of some predictors. The analyses of women with either a diagnosis of or treatment for PP-PTSD in the past, or who currently had symptoms, were therefore done to decrease the chance of missing women who had recovered from their earlier PP-PTSD symptoms. Lastly, there could be some form of selection bias by excluding an extra 584 participants from the study for this article, because they did not complete the entire questionnaire. These excluded participants less often reported a threat to their own life, actual or threatened serious injury and a threat to their physical integrity compared to the 1599 women included in the analyses in the current article, which could have led to an overestimation of the effect of the predictors.

There are also several strengths to this study. First and foremost, it was possible to illicit a large response through an online questionnaire, which makes this study larger than any of the previous studies about the occurrence of PP-PTSD in women with a traumatic childbirth experience (7, 10). Also, the questionnaire contained four psychological measurement tools, which have been validated for measuring coping abilities, social support, fear of childbirth and symptoms of PTSD. In addition, the study population differs significantly from the general Dutch population with regard to parity at the time of the traumatic childbirth experience, age of the mother during childbirth, gestational age, ethnicity, mode of delivery, and responsible caregiver during pregnancy and delivery^2^,^3^. These variables correspond to the risk factors known in literature, which increases the assumption that the study population is a representative group of women with a traumatic childbirth experience (7, 10). Finally, to our knowledge, this study is the first exploratory study that tried to establish predictive factors and determine their respective weight in predicting PP-PTSD according to the DSM-5 in women with a traumatic childbirth experience, thereby creating new insights into the role of antepartum vulnerability factors with regard to the development of PP-PTSD symptoms in women with a traumatic childbirth experience. These new insights could be the basis for further research into interventions aimed at preventing PP-PTSD.

## Conclusion

This study identified several antepartum and intrapartum vulnerability factors in women with a self-reported traumatic childbirth experience that were predictive for the development of postpartum PTSD symptoms, corresponding with criteria B, C, D, and E in the DSM-5. Four significant contributing factors predictive of developing PP-PTSD emerged from this study: lack of social support, low sense of coherence, experiencing “threatened death” and experiencing “actual or threatened injury to the baby.” This predictive model had an overall accuracy of 66.5%, a sensitivity of 80.8%, and specificity of 62.6%. Despite the retrospective method used for this study, this predictive model demonstrates the importance of coping abilities and social support in women reporting PP-PTSD symptoms after a traumatic childbirth experience. Further research should focus on the effect of interventions during pregnancy aimed at strengthening coping skills and increasing social support in pregnant women.

## Author contributions

MH and CS designed the study and collected the data. MvP and JvD provided feedback on the study design. MvH performed the data analysis and drafted the manuscript. MH, CS, JvD, and MvP critically revised the manuscript. All authors approved the final version of the paper.

## Conflict of interest statement

The authors declare that the research was conducted in the absence of any commercial or financial relationships that could be construed as a potential conflict of interest.

## Footnotes

^1^Survey Monkey: an Online Survey Tool [Internet]. San Mateo: Survey Monkey. Available online at: https://nl.surveymonkey.com/ (Accessed Jan 10, 2018).

^2^Perinatal care in the Netherlands 2013 [Internet]. Foundation Perinatal Registration The Netherlands (2014).

^3^CBS, (Central Bureau for Statistics). Geboorte; kerncijfers. Den Haag/ Heerlen2017.
